# Unveiling detoxifying symbiosis and dietary influence on the Southern green shield bug microbiota

**DOI:** 10.1093/femsec/fiae150

**Published:** 2024-11-07

**Authors:** Magda A Rogowska-van der Molen, Hristina V Savova, Elke A T Janssen, Theo van Alen, Silvia Coolen, Robert S Jansen, Cornelia U Welte

**Affiliations:** Department of Microbiology, Radboud Institute for Biological and Environmental Sciences (RIBES), Radboud University, PO Box 9010, 6500 GL Nijmegen, The Netherlands; Department of Microbiology, Radboud Institute for Biological and Environmental Sciences (RIBES), Radboud University, PO Box 9010, 6500 GL Nijmegen, The Netherlands; Department of Microbiology, Radboud Institute for Biological and Environmental Sciences (RIBES), Radboud University, PO Box 9010, 6500 GL Nijmegen, The Netherlands; Department of Microbiology, Radboud Institute for Biological and Environmental Sciences (RIBES), Radboud University, PO Box 9010, 6500 GL Nijmegen, The Netherlands; Department of Microbiology, Radboud Institute for Biological and Environmental Sciences (RIBES), Radboud University, PO Box 9010, 6500 GL Nijmegen, The Netherlands; Translational Plant Biology, Department of Biology, Faculty of Science, Utrecht University, P.Box 800.56, 3508 TB Utrecht, The Netherlands; Department of Microbiology, Radboud Institute for Biological and Environmental Sciences (RIBES), Radboud University, PO Box 9010, 6500 GL Nijmegen, The Netherlands; Department of Microbiology, Radboud Institute for Biological and Environmental Sciences (RIBES), Radboud University, PO Box 9010, 6500 GL Nijmegen, The Netherlands

**Keywords:** insect–plant–microbe interactions, plant defences, detoxification, symbionts, nitronate monooxygenase, 3-nitropropionic acid

## Abstract

The Southern green shield bug, *Nezara viridula*, is an invasive piercing and sucking pest insect that feeds on crops and poses a threat to global food production. Insects live in close relationships with microorganisms providing their host with unique capabilities, such as resistance to toxic plant metabolites. In this study, we investigated the resistance to and detoxification of the plant metabolite 3-nitropropionic acid (NPA) by core and transient members of the *N. viridula* microbial community. Microbial community members showed a different tolerance to the toxin and we determined that six out of eight strains detoxified NPA. Additionally, we determined that NPA might interfere with the biosynthesis and transport of l-leucine. Moreover, our study explored the influence of diet on the gut microbial composition of *N. viridula*, demonstrating that switching to a single-plant diet shifts the abundance of core microbes. In line with this, testing pairwise microbial interactions revealed that core microbiota members support each other and repress the growth of transient microorganisms. With this work, we provide novel insights into the factors shaping the insect gut microbial communities and demonstrate that *N. viridula* harbours many toxin-degrading bacteria that could support its resistance to plant defences.

## Teaser

Core microbial community members from the gut intestinal tract of the Southern green shield bug support each other’s growth and some are able to degrade toxic plant metabolites.

## Introduction

The Southern green shield bug (*Nezara viridula*) is a piercing and sucking insect that feeds on plant sap causing wilting and silver damage (Todd 1989, Lucini and Panizzi 2018, Medina et al. 2018). This pest insect is a global threat to diverse crops from the Fabaceae, Solanaceae, and Brassicaceae families, which comprise economically and nutritionally important crops like soybean, tomato, or cabbage. With the increasing global demand for food, decreasing crop losses has become an urgent challenge. Despite many efforts to decrease the number of pest insects like *N. viridula*, traditional pest control strategies with chemical insecticides have had a limited effect. Moreover, these chemical insecticides have a pronounced effect on human health and contribute to environmental pollution (Costa et al. 2007, Botías et al. 2019). Therefore, an effective and sustainable solution is needed to secure food production in the future. Recent studies demonstrated that targeting symbiotic microorganisms could be an alternative solution to decrease crop losses since gut microbiota support insect resistance against toxic compounds and insecticides (van den Bosch and Welte 2017, Chung et al. 2018, Itoh et al. 2018, Medina et al. 2018, Rogowska-van der Molen et al. 2023). While this is a promising approach to the development of sustainable pest management strategies, fundamental knowledge of the tritrophic interactions between pest insects, their microbiota, and host plants is lacking.

The insect microbiome consists of core and transient microbiota. Core microbiota are obligate and facultative symbionts that are relatively stable and regularly associated with the host, while the transient microbiota represents microbes that inhabit the host only for a rather short period. Although transient microbes only constitute a small proportion of the microbiome (<1%), a recent study on mosquitoes showed that both core symbionts and transient microbes strongly affect insect reproductive cycles via the provision of B vitamins (Serrato-Salas and Gendrin 2023). Therefore, transient microbes could be potentially as important as core symbionts. Understanding the interplay between core and transient microbiota members is thus crucial for comprehending the function of the insect microbial community.

Several components determine insect microbiota composition, such as diet, host phylogeny, and microbe–microbe interactions (Colman et al. 2012, Amato et al. 2019). To sustain growth, microbes that live in consortia, such as those in the insect gut, might depend on each other by biosynthesizing and exchanging certain metabolites (Zhang et al. 2018). The gut bacterial community of evolutionarily distinct insects is similar during feeding on the same diet. Huang et al. (2021) determined that gut microbes facilitate convergent evolution during the adaptation to the same diet in phylogenetically distinct species. Microbes seem to not only have an impact on insect viability and development but also an adaptations to changing environments (van den Bosch and Welte 2017, Itoh et al. 2018, Sato et al. 2021). One adaptation is evading plant defences by detoxifying phytochemicals (Bruce 2015). A recent study indicated that potato tuber moth microbiota mediated detoxification of α-chaconine and α-solanine by harbouring gene clusters involving α-rhamnosidase, β-glucosidase, and β-galactosidase (Wang et al. 2022) and cabbage root fly larvae microbiota contributed to 2-phenylethyl isothiocyanate degradation by harbouring isothiocyanate hydrolase enzymes in plasmids (Welte et al. 2016a,b).

*Nezara viridula*’s core microbiota can degrade isoflavonoids and deactivate soybean protease inhibitors, potentially facilitating its resistance to plant chemical defences (Medina et al. 2018). However, the role of transient microbiota remains elusive. Besides isoflavonoids, leguminous plants biosynthesize other defence compounds, such as 3-nitropropionic acid (NPA), to deter insects. When ingested by mammals, NPA causes irreversible inhibition of the mitochondrial electron transport chain and succinate dehydrogenase in the tricarboxylic acid cycle (TCA), causing acute toxicity (Francis et al. 2013, Anderson et al. 2016). Several soil- and gut-associated bacteria degrade NPA using either propionate-3-nitronate monooxygenase (P3N), nitronate monooxygenase (NMO), or putative nitronate monooxygenase (PNMR), encoded by *pno*A, *nmo*A, or *pnm*R, respectively (Anderson et al. 2000, Nishino et al. 2010, Rogowska-van der Molen et al. 2022). A recent study demonstrated that *N. viridula* feeding on the NPA-rich legume vine crown vetch (*Securigera varia*) showed no symptoms of intoxication, and the isolated gut microbe *Pseudomonas* sp. Nvir was able to detoxify NPA releasing CO_2_, nitrite, and nitrate (Rogowska-van der Molen et al. 2022). Given *N. viridula*’s apparent resistance to NPA, we studied the possible involvement of core and transient gut-associated microbiota in NPA degradation.

To investigate the mechanistic role of the *N. viridula* gut microbiota on the toxin resistance of its host, we investigated previously identified core members and newly isolated transient gut microbiota by first evaluating the presence of detoxifying genes in genomes and plasmids with genome sequencing. Additionally, we cultivated eight *Nezara*-associated bacterial isolates to assess their resistance and ability to metabolize NPA. These analyses indicated that bacteria had a different resistance pattern to NPA even though most were capable of NPA detoxification and encoded either *pno*A, *nmo*A, or *pnm*R in their genomes. Transcriptomic and metabolomic analyses of the obligate symbiont *Pantoea* sp. Nvir suggested a possible negative influence of NPA on amino acid metabolism and transport. ITo assess whether diet plays a role in altering bacterial community, insects were fed for 6 months with either black mustard (Brassicaceae) or black nightshade (Solanaceae) plants and afterwards, their gut microbiota was compared to insects feeding from multiple plants. Our findings indicated that switching to a single-plant-based diet shifted the relative abundance of the gut core microbiota of *N. viridula*. Experiments showed that core microbiota had many mutualistic interactions and suppressed the growth of transient bacteria. We provide insights into the influence of dietary components on the insect gut microbial community composition and show that *N. viridula* harbours many toxin-degrading bacteria that support its resistance to plant defence metabolites.

## Materials and methods

### Insect collection and rearing

*Nezara viridula* were collected from creeping thistle (*Cirsium arvense*) in the Netherlands (51.348028, 6.128802) and were provided by Wageningen Plant Research (Bleiswijk, the Netherlands) in March 2023. The insects were transferred to a greenhouse and placed in a rearing cage to establish a colony (native population). *Nezara viridula* individuals were reared in a greenhouse facility with normal daylight and additional light to obtain a photoperiod of 16:8 h (light:dark) year-round. Insects were provided with sunflower (*Helianthus annuus*), soybean (*Glycine max*), brown mustard (*Brassica juncea*) seeds, flat beans (*Phaseolus vulgaris*), and the native plants crown vetch (*S. varia*), black mustard (*Brassica nigra*), and black nightshade (*Solanum nigrum*).

### Insect dissection and pure culture isolation

Five complete gut systems (M1–M4 sections) and salivary glands of adult *N. viridula* were dissected directly after submersion of insects in 70% ethanol for ~1 min after which movement stopped. Dissection was performed under nonsterile conditions using a stereomicroscope, scalpel, and forceps. Separation of tissues from the insect body was performed in phosphate-buffered saline solution (PBS; 137 mM NaCl, 2.7 mM KCl, 10 mM Na_2_HPO_4_, and 1.8 mM KH_2_PO_4_, pH 7.4) to prevent rupture of the delicate tissue.

For culturing and isolation, salivary glands and complete gut systems were disrupted in 200 µl PBS by vortexing. Saliva and frass samples were collected from adult *N. viridula* as described previously (Coolen et al. 2024). Briefly, *Pantoea, Bacillus frigoritolerans, Bacillus megaterium, Klebsiella pneumoniae*, and *Pseudomonas* sp. Nvir were isolated from the gut systems, *Sodalis* from salivary glands and both *Serratia* strains from frass. Although sequencing data showed the presence of many bacterial species associated with *N. viridula*, we were not able to isolate and culture any other strains.

Next, samples were diluted and incubated in M9 mineral salt medium [33.7 mM Na_2_HPO_4_, 22 mM KH_2_PO_4_, 8.55 mM NaCl, 9.35 mM NH_4_Cl, 1 mM MgSO_4_, 0.3 mM CaCl_2_, Thauer vitamin mixture (Sowers and Ferry 1985), trace elements (Kurth et al. 2019), pH 7.2] containing either 100 µM of α-solanine or NPA as a carbon source or plated on Luria–Bertani (LB) agar (0.5% peptone, 0.3% yeast extract, 0.5% NaCl, and 1.5% agar) or mannitol agar (2.5% *n*-mannitol, 0.5% yeast extract, 0.3% peptone, and 1.5% agar) plates without toxins. Agar plates were incubated at 30°C for a maximum of 3 weeks after which single colonies were transferred to new LB agar plates at least six times before they were considered pure. Liquid cultures were incubated at 30°C at 200 r/m for 2 weeks. All isolated strains were ultimately plated on M9 mineral salt medium agar plates containing either 100 µM of α-solanine or NPA, which served as carbon source and incubated at 30°C for 1 week. Colonies were transferred to new M9 agar plates containing either 100 µM of α-solanine or NPA at least six times before they were considered pure.

### Dietary influence on microbiota composition

Fifteen *N. viridula* (first to fifth instars and adults) individuals were collected in May 2023 from the native population and placed in separate rearing cages that contained either black mustard or black nightshade plants. Insects were reared for 6 months and afterwards, three *N. viridula* adults were collected from black mustard, black nightshade, and native population (control), and dissected. DNA was extracted from complete gut systems (M1–M4 sections and hindgut) for determination of gut microbial profile with 16S rRNA gene amplicon sequencing.

As a control, to determine the plant microbiome, noninfested black mustard and black nightshade plants (5 g each) were harvested and washed for 30 min in PBS solution (137 mM NaCl, 2.7 mM KCl, 10 mM Na_2_HPO_4_, and 1.8 mM KH_2_PO_4_, pH 7.4) with six 1-min vortex periods. Tubes were spun down for 15 min at 3200 r/m and pellets were used for DNA isolation.

### DNA isolation, genome sequencing, and data analysis

DNA was isolated from all pure cultures, dissected gut and plant, using a DNeasy PowerSoil Kit (QIAGEN, the Netherlands) according to the manufacturer’s protocol. For pure cultures, 1 ml of pelleted cells were lysed with the TissueLyser LT (QIAGEN) for 2 min at 50 Hz. MiSeq sequencing (Illumina, San Diego, CA, USA) was performed with the Nextera XT Library Preparation Kit (Illumina) according to the manufacturer’s instructions. Paired-end sequencing (2 × 300 bp) was performed using the Illumina MiSeq sequencer (Illumina) and the MiSeq Reagent Kit v3 (Illumina) according to the manufacturer’s protocol. Long-read sequencing was performed with the MinION R9 flow cell (FLO-MIN106; Nanopore, Oxford, UK), according to the manufacturer’s protocol, using unsheared DNA (no size selection) and NEBNext formalin-fixed, paraffin-embedded repair mix (M6630), a ligation sequencing kit (SQK-LSK109), the NEBNext end repair/dA-tailing module (E7546), a flow cell priming kit (EXP-FLP001), NEB Blunt/TA ligase master mix (M0367), the NEBNext quick ligation module E6056, and barcode kit EXPNBD104. The libraries were checked for quality and size distribution using the Agilent 2100 Bioanalyzer and the High sensitivity DNA kit (Agilent Technologies, Santa Clara, CA, USA). Quantitation of the libraries was performed by the Qubit dsDNA HS Assay Kit (Thermo Fisher Scientific Inc., Waltham, USA).

Oxford Nanopore reads were trimmed to remove adapters using Porechop (0.2.3_seqan2.1.1) (Wick et al. 2017a) and assembled with Flye (2.9) (Kolmogorov et al. 2019). Sequences were aligned with Minimap2 (2.22) (Li 2018) and consensus assemblies were generated with Racon (v1.4.20) (Vaser et al. 2017). Quality trimming and filtering of MiSeq Illumina reads was performed with BBDuk (BBTools v38.75) (Bushnell 2014) and extracted reads were aligned with BBMap (v38.75). Genomes were assembled using MiSeq Illumina and Nanopore reads with Unicycler (v.0.4.4) (Wick et al. 2017b). The taxonomy and the quality of the generated genomes were assessed through a single-copy marker gene analysis CheckM (1.1.2) (Parks et al. 2015). Genomes were annotated with Prokka (1.13.4) (Seemann 2014). Metabolic pathways were predicted with KEGG (Kanehisa and Goto 2000).

### 16S rRNA gene amplicon sequencing and analysis

The gut bacterial community of *N. viridula* was determined by amplification of the V3–V4 region of the 16S rRNA gene. The sequencing was performed by Macrogen (the Netherlands) with *Bac341F* and *Bac806R* primers (Herlemann et al. 2011, Caporaso et al. 2012) using an Illumina MiSeq sequencer (Illumina). Paired-end (2 × 301 bp) reads libraries were prepared with the Herculase II Fusion DNA Polymerase Nextera XT Index Kit V2 (Illumina).

The quality of the raw paired-end sequences was checked with FastQC 0.11.8 (Bushnell 2014). The reads were then filtered, and adapters were trimmed. Approximately 40 000–70 000 paired-end sequencing reads were obtained per sample. The data were further processed using the DADA2 1.8 pipeline (Callahan et al. 2016) in R. Phylogenetic taxonomy of the reads was assigned using the SILVA 16S rRNA database 138.1 (Quast et al. 2012). Count data were normalized to relative abundance. Microbial community profiles were analysed and amplicon sequence variants (ASVs) were visualized using the phyloseq (McMurdie and Holmes 2013) and ggplot2 (Wickham 2009) packages in R.

### RNA isolation and transcriptome sequencing

*Pantoea* sp. Nvir was cultured in 150 ml M9 mineral salt medium supplemented with glucose 0.08% for 24 h. Upon glucose depletion, either 100 µM of NPA (treated) or glycerol (control) was supplemented to the culture. Biomass samples for RNA extraction were taken 2 h after the supplementation. Samples (5 ml) were centrifuged at 3500 × *g* for 10 min at 4°C and RNA was extracted with the RiboPure™-Bacteria Kit (Thermo Fisher Scientific) according to the manufacturer’s instructions. Prior to sequencing, bacterial mRNA was purified with the MICROBExpress™ Bacterial mRNA Enrichment Kit (Thermo Fisher Scientific) according to the manufacturer’s instructions. Transcriptome libraries were constructed using the TruSeq® Stranded mRNA Library Prep protocol (Illumina) according to the manufacturer’s instructions. Obtained libraries were checked with Qubit as described before. Equimolar pooled libraries were sequenced using the Illumina MiSeq sequencer (Illumina). For sequencing, the 150 bp sequence chemistry was performed using the MiSeq Reagent Kit v3 (Illumina) according to the manufacturer’s protocol in one direction. For both control and treated cultures, RNA was extracted from three independent biological replicates.

The quality of Illumina single-end transcriptome raw reads was assessed with FASTQC (0.11.8) (Bushnell 2014) prior to reads mapping. Single-end reads were mapped against the annotated genome using Kallisto (0.46.2) (Bray et al. 2016) and outputs were summarized with MultiQC 1.11 (Ewels et al. 2016). Read counts were transformed in Tximport (Soneson et al. 2015) for differential gene expression analysis using BioConductor packages in R environment (Huber et al. 2015). To determine differentially expressed genes log2-fold change cut-off of 2 was set. In addition, to address the issue of multiple testing, a false discovery rate correction was performed with a *P* < .05 value using the Benjamini–Hochberg method (Benjamini and Hochberg 1995).

### 3-nitropropionic degradation assay

The axenic cultures of bacterial isolates were pregrown aerobically in batch (50 ml; *n* = 3) for 24 h (30°C, 200 r/m) in M9 mineral salt medium as described earlier, until OD_600_ of 0.7 ± 0.1. Culturing was performed under aerobic conditions because anaerobic culturing led to comparable results (Coolen et al. 2024). Hereafter, 100 µM of NPA was supplemented to the pregrown culture. For the determination of NPA, nitrite (NO_2_^−^), and nitrate (NO_3_^−^) concentrations, 1 ml samples were collected for 24 h. The supernatant was collected after centrifugation at 20 000 × *g* for 3 min and was stored at −20°C until analysis.

High-performance liquid chromatography (HPLC) was used to determine NPA concentrations. Using an Agilent 1100 Series LC system equipped with a diode array detector and a C18 column [LiChrospher 100 RP-18 end-capped (5 µm) column, 125 mm × 4 mm, Merck], gradient analysis was performed with a flow rate of 1.2 ml min^−1^ using 0.1% orthophosphoric acid in water (Solvent A) and acetonitrile (Solvent B). The gradient analysis was performed with 0% Solvent B at 0–5 min, 10% Solvent B at 5–6 min, 45% Solvent B at 6–8 min, 85% Solvent B at 8–10 min, 90% Solvent B at 10–12 min, 90% Solvent B at 12–13 min, and 0% Solvent B at 13–15 min. Before the analysis, 200 µl of supernatant was acidified with 25 µl 1 M sulphuric acid, after which 100 µl of the sample was injected. NPA was measured at 210 nm with a retention time of 2.9 min.

To determine nitrite (NO_2_^−^) and nitrate (NO_3_^−^) concentrations, a Griess-reagent assay was used with standard curves of NaNO_3_ and NaNO_2_. The supernatant (100 µl) collected during the NPA degradation assay was transferred to a 96-well plate containing 100 µl of Griess reagent [50 µl of reagent A: 1% (w/v) sulfanilic acid in 1 M HCl and 50 µl of reagent B: 0.1% (w/v) naphthyl ethylene diamine dihydrochloride in demineralized water]. The plate was incubated for 10 min at room temperature and absorbance was measured at 540 nm with a microplate reader to determine nitrite (SpectraMax 190, Molecular Devices, San Jose, CA, USA). Next, 27 µl of VCl_3_ (10 mg ml^−1^ in 1 M HCl) was added to the sample to reduce nitrate to nitrite. Subsequently, the 96-well plate was incubated in the dark at 60°C for 30 min, whereafter absorbance was measured at 540 nm.

### LC-qToF-MS untargeted metabolomics

To determine the metabolome of *Pantoea* sp. Nvir under NPA-supplemented conditions, metabolites were extracted from a 5 ml sample (*n* = 4; treated and control samples) obtained after 6 h of NPA supplementation as described under the NPA degradation assay section. Cell pellets were weighed and equivalent amounts of ~500 µl acetonitrile:methanol:H_2_O (40:40:20; v:v:v) were added to each sample and vortexed vigorously for 10 s. Samples were incubated for 5 min on ice and subsequently centrifuged at 20 000 × *g* for 5 min. The supernatant was transferred into a new tube and stored at −70°C until metabolite analysis.

Samples were analysed with a 1290 Infinity II LC system coupled to a 6546 Q-ToF MS (Agilent Technologies) (LC/Q-ToF-MS) as described (Jansen et al. 2020). In short, a 2 µl sample was injected onto a Diamond Hydride Type C column (Cogent) and separated using a 0.4 ml min^−1^ gradient of water with 0.2% formic acid (A) and acetonitrile with 0.2% formic acid (B). The gradient was as follows: 0–2 min: 85% B, 3–5 min: 80% B, 6–7 min: 75% B, 8–9 min: 70% B, 10–11 min: 50% B, 11–14: 20% B, 14–24: 5% B, followed by 10 min re-equilibration at 85% B. Detection of compounds was performed from m/z 50–1200 in the positive and negative ionization modes. Raw data were converted to mzXML format using ProteoWizard software (Chambers et al. 2012) and two sample groups were compared using XCMS online with centWave method and set parameters (15 ppm, 10–120 peak width, Signal/Noise threshold 6), and obiwarp retention time correction (profStep 1). The peak table was filtered for features that were more abundant in the NPA-treated samples compared to controls (*P* < .01, max intensity > 10^6^). From the resulting list, four features from positive and ten from negative ionization modes, respectively, were further analysed. For identification, MS/MS fragmentation spectra were obtained using the LC-qToF-MS method described above, operated at a collision energy of 10, 20, and 40 V. For metabolite annotation, fragmentation spectra were queried in the MassBank and MoNA databases (August 2023) (MoNA—MassBank of North America 2007, Horai et al. 2010). To validate the putative identification of 2-isopropylmalate, a chemical standard was obtained (>98%; Sigma Aldrich, the Netherlands), followed by a comparison of the fragmentation spectra between *Pantoea* sp. Nvir metabolite and standard compound. A mirror plot was generated with a Spectra package in R with a match tolerance of 0.1 ppm.

To identify the NPA influence on the l-leucine biosynthesis pathway, the relative abundance of 3-hydroxy-3-methyl-2-oxobutanoate, 2-oxoisovalerate, 2-isopropylmalate (putatively annotated based on accurate mass), and l-leucine was determined using Qualitative Analysis 10.0 (Agilent Technologies).

### Determination of resistance against NPA

Resistance of microbial isolates against NPA was evaluated with disc diffusion assays. Bacterial cultures were pregrown in LB medium (30°C, 200 r/m) for 24 or 168 h (for *Sodalis* sp. Nvir) and subsequently diluted to OD_600_ of 0.1 ± 0.05. An aliquot of 100 µl of the culture was plated on LB agar plates (*n* = 2). A paper disc (6 mm diameter, Whatman) soaked with 10 µl of NPA solution (0, 93.75, 187.5, 375, 750, and 1000 mM, dissolved in dimethylsulfoxide) was placed on the plate. Plates were incubated for 48 h at 30°C and afterwards, the diameter of inhibition was measured.

### Pairwise species interactions on phloem-sap medium plates

The growth of bacterial pure cultures in phloem-sap (PS) medium [100 mM l-serine, 100 mM methionine, and 38.5 mM aspartic acid (pH = 7.0, adjusted with NaOH)] (Tan et al. 2016) was determined in varying sucrose concentrations [0.5%, 1%, 2%, 5%, 10%, and 20% (v/v) sucrose] by measuring the OD_600_ for 114 h in a microplate reader (SpectraMax 190, Molecular Devices). Cultures were pregrown for 24 or 168 h (for *Sodalis* sp. Nvir) in LB medium (30°C, 200 r/m), and accordingly diluted to an OD_600_ of 0.05 and inoculated in a 96-well plate to a well containing 200 µl PS medium (*n =* 3). Optical density was measured in 15-min intervals. According to the growth curve, PS medium with 10% sucrose solution was favoured by all bacterial isolates and therefore this sucrose concentration was used further on to determine pairwise species interactions on PS agar plates.

The pairwise (binary) microbial interactions were assessed in 10% sucrose PS agar plates (1.5% agar) containing 0.03 g l^−1^ bromocresol green as the stain. Per background species, an equivalent volume needed for OD_600_ = 5 was collected by centrifugation at 16 000 × *g* at 3 min. Cell pellets were resuspended in 150 µl of 0.9% NaCl and spread on PS agar using sterile glass beads (*n* = 2). Plates were incubated for 2 h at 30°C before pinning to provide growth advance to the background species. Query (pinned) species were prepared by centrifugation (16 000 × *g*, 3 min) of 2 ml cultures and pellets were resuspended in 300 µl 0.9% NaCl. After a 2 h incubation period, using a multichannel pipette, 5 µl of the query species was pinned on top of the background species (*n =* 4). In addition, plates with no background species were prepared and pinned as described earlier (*n* = 4). The plates were incubated for 48 h at 30°C and the plates were imaged using Samsung Galaxy S22 Ultra 108-megapixel camera. The colony surface area was determined using ImageJ (version 2.35). The growth effects were classified as positive, negative, or neutral if the colony surface area was significantly (two-sided *t*-test, *P-*value cut-off = .05) larger, smaller, or similar in comparison with plates without background species. Based on this, the type of interaction between two species was given, based on the reciprocal effects, namely mutualism, commensalism, amensalism, parasitism, and competition.

## Results

### Genomic and functional characterization of *N. viridula* microbiota reveals nutrient production and detoxification abilities

*Nezara viridula* core microbiota consists of four bacterial genera, namely *Pantoea, Sodalis, Serratia*, and *Commensalibacter*, representing >99% of the gut microbial community (Coolen et al. 2024). The remaining fraction represents a low abundant transient microbial community. In this study, we sequenced previously obtained pure cultures (Coolen et al. 2024) and new species isolated from the gut, frass, and saliva of *N. viridula*. The genomic characteristics of core and transient *N. viridula* microbiota are summarized in Table 1. Core [*Pantoea* sp. Nvir, *Serratia marcescens* (S-F1) and (S-F5), *Sodalis* sp. Nvir] and transient [*B. megaterium* (S-ITC1), *B. frigoritolerans* (S-Sol1), *K. pneumoniae*, and *Pseudomonas* sp. Nvir] microbiota members showed differences in genome size, number of plasmids, and GC content. Genomes of core bacteria were large with a GC content of 55%–60%, and harboured multiple plasmids. Of all bacteria belonging to the core microbiota, *Pantoea* sp. Nvir had the smallest genome, but the largest plasmid with 508 307 bp. Plasmids A and B of Pantoea contained genes encoding carbohydrate-active and plant metabolite detoxification enzymes (quercetin 2,3-dioxygenase, phenolic acid decarboxylase and putative tartrate decarboxylase). Both *S. marcescens* strains had similar genome sizes and numbers of coding sequences (CDS). The analysis also showed that *Sodalis* sp. Nvir, known to colonize salivary glands nearly entirely, had a large genome that included genes for flagellar assembly indicating the ability to adapt to a free-living lifestyle. Transient microbiota representatives had large genomes and divergent GC content ranging from 37% to 62%. Unlike *B. megaterium* and *Pseudomonas*, only *B. frigoritolerans* and *K. pneumoniae* had four and one plasmids, respectively. Overall, the genomes and plasmids of core and transient microbes were similar in size and number of CDS. Further functional analysis of symbiosis-associated genes could unveil their role in *N. viridula* gut.

**Table 1.** Summary of *N. viridula* core and transient microbiota genomic characteristics.

	Species	Type of DNA	Size (bp)	Number of segments	Status	GC (%)	Completeness (%)	Contamination (%)	CDS	rRNA	tRNA
Core microbiota	*Pantoea* sp. Nvir	Genome	3 954 582	1	Circular	59.0	99.99	0.66	3507	22	82
		Plasmid A	508 307	1	Circular	57.3			462		
		Plasmid B	200 700	1	Circular	56.1			183		
		Plasmid C	50 023	1	Circular	51.8			51		
	*S. marcescens* (S-F1)	Genome	5 201 660	1	Circular	59.9	99.8	0	4783	22	93
		Plasmid A	148 677	1	Circular	52.6			162		
		Plasmid B	105 264	1	Circular	53.6			110		
		Plasmid C	100 281	1	Circular	51.1			108		
	*S. marcescens* (S-F5)	Genome	5 201 368	1	Circular	59.9	99.8	0	4783	22	91
		Plasmid A	105 712	1	Circular	52.8			102		
		Plasmid B	105 264	1	Circular	53.6			110		
	*Sodalis* sp. Nvir	Genome	5 497 650	44	Contigs	55.5	97.62	0	7519	21	67
Transient microbiota	*B. megaterium* (S-ITC1)	Genome	6 115 434	235	Contigs	37.3	99.43	1.87	6175	42	140
	*B. frigoritolerans* (S*-*Sol1)	Genome	5 646 898	1	Circular	40.6	100	0	5410	39	77
		Plasmid A	248 967	1	Circular	36.8			259		
		Plasmid B	143 153	1	Circular	37.7			133		
		Plasmid C	96 428	1	Circular	36.4			108		
		Plasmid D	16 027	1	Circular	36.5			15		
	*K. pneumoniae*	Genome	5 400 374	1	Circular	57.6	100	0.43	4947	25	87
		Plasmid A	281 938	1	Circular	52.8			280		
	*Pseudomonas* sp. Nvir	Genome	5 856 348	1	Circular	62.1	99.61	3.92	5414	22	78

After determining the basic genomic and taxonomic characteristics, we investigated the microbial nutrient production potential and found that all bacteria associated with the core microbiota as well as *Klebsiella* from the transient microbiota had the genomic potential to biosynthesize all essential amino acids, namely valine, leucine, isoleucine, threonine, methionine, arginine, lysine, histidine, phenylalanine, and tryptophan. Bacteria of both core and transient microbiota could additionally synthesize vitamins B1, B2, B5, B6, and B7, based on their genomic potential (Table S1). Since many insects benefit from symbiotic microorganisms to detoxify phytotoxins and pesticides (van den Bosch and Welte 2017, Itoh et al. 2018), we inspected the potential of *N. viridula* microbiota to degrade toxins present in the diet of *N. viridula*. In our rearing facility, insects feed on black mustard, black nightshade, and crown vetch which synthesize isothiocyanates (ITC), solanaceous glycoalkaloids, and NPA, respectively to deter insects (Fahey et al. 2001, Mohy-ud-Din et al. 2010, Al-Snafi 2016). A previous study on the gut microbiome of cabbage root fly demonstrated that *sax*A plasmid gene coding for isothiocyanate hydrolase responsible for the degradation of 2-phenylethyl isothiocyanate (Welte et al. 2016b), but none of the *N. viridula*’s microbiota had *sax*A on plasmids or genome. However several microbes harboured genes encoded in the genomes involved in the sugar and toxin metabolism, namely β-glucosidase (*bgl*B) and β-galactosidase (*lac*Z, *ebg*A, *bga*A, and *cbg*A; Table S2). As shown earlier, these genes are involved in the partial degradation of α-chaconine and α-solanine (Hennessy et al. 2018, 2020). To metabolize NPA, either *pno*A, *nmo*A, or *pnm*R encoding for P3N and NMOs, respectively, must be present (Rogowska-van der Molen et al. 2022). Seven members of the *N. viridula* microbiota possessed at least one of those genes, however all were present in the genome. This suggests that *N. viridula*-associated gut bacteria may have undergone an environmental selection and thus confer a long-term adaptive response to NPA. On the other hand, *Sodalis* sp. Nvir seemed to be the only one lacking the inferred genomic potential to detoxify NPA.

Due to the widespread occurrence of NPA-degrading genes among the *N. viridula* microbiota, we analysed genes’ phylogeny by performing a multiple sequence alignment of *pno*A, *nmo*A and *pnm*R amino acid sequences (Fig. S1B). The enzymes encoded by *nmo*A and *pno*A showed substantial similarity with a tendency to cluster together, and PnmR was markedly different. Also, genus-dependent clustering of NmoA and PnoA sequences was noticed in *Serratia* and *Bacillus. Pseudomonas* sp. Nvir harboured both *pno*A and *pnm*R but only the latter was transcribed during culturing with NPA (Rogowska-van der Molen et al. 2022). This underlines that the presence of a gene alone does not demonstrate biodegradation activity. As most bacteria from both core and transient microbiota contained genes potentially enabling them to degrade NPA, we proceeded with their functional characterization of NPA resistance and degradation by the eight microbial isolates.

### *Nezara viridula* microbiota detoxify NPA *in vitro* with variable NPA tolerance

**Figure 1.**
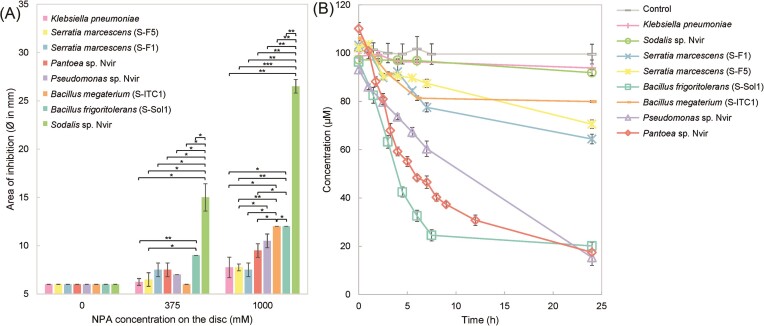
Characterization of bacteria isolated from the *N. viridula* microbiome to resist and detoxify NPA. (A) Bacterial gut isolates were tested for their resistance to NPA in disc diffusion assays with 10 µl of increasing NPA concentration (0–1000 mM) applied to the paper discs (6 mm Ø; Fig. S2 and Table S3). The diameter of the inhibition zone is expressed in mm ± standard deviation (biological duplicates). Significance was tested with a Student’s *t*-test for unpaired samples and accepted at *P* < .05 (*), < .01 (**), and < .001 (***). The minimal experimental area of inhibition is 6 mm, which is due to the paper disc diameter of 6 mm. (B) Bacterial gut isolates were incubated in 50 ml M9 mineral salt medium supplemented with 100 µM NPA and detoxification was monitored for 24 h. Control represents uninoculated M9 medium containing NPA. NPA concentrations were measured by HPLC. Data are represented as mean ± standard error (biological triplicates).

The isolated microbial community members of the *N. viridula* gut microbiota were assessed for resistance against NPA with disc diffusion assays (Fig. 1A). *Klebsiella* and the two *Serratia* strains were most resistant and showed almost no inhibition (<8 mm Ø zone of inhibition around the 6 mm Ø paper disc) even at the highest NPA concentration. *Pantoea, Pseudomonas*, and two *Bacillus* strains were less resistant to NPA and showed a diameter of inhibition varying from 9 to 12 mm. The most susceptible strain was *Sodalis*, whose diameter of inhibition at 1000 mM NPA reached 27 mm. Combining these results with the genomic analysis revealed that strains, which harboured genes encoding for NPA-degrading enzymes were substantially more resistant to NPA than *Sodalis* which did not carry them.

To test whether the observed NPA resistance could be caused by degradation, we investigated whether members of the microbiota obtained from *N. viridula* detoxify NPA in cultures. All isolates were cultured in liquid M9 minimal salt medium supplemented with 100 µM NPA (Fig. 1B). *Pantoea, Pseudomonas*, and *B. frigoritolerans* demonstrated rapid NPA degradation within 24 h, whereas both *Serratia* strains *and B. megaterium* only partially degraded NPA, leaving more than half of the NPA in the medium after 24 h. All NPA-degrading strains exhibited prompt degradation in the beginning, followed by substantially reduced degradation after 8–10 h. These observations might be the result of the accumulation of inhibitory by-products like nitrite and nitrate in the culture medium (Figs S3A and B, S5). While our genomic analysis indicated that *Klebsiella* harboured *pno*A and therefore has the potential to metabolize NPA, NPA degradation was not observed under the culturing conditions chosen in this study. *Sodalis* did not exhibit the capability to degrade NPA either, as expected from the genomic predictions. Altogether, our genomic analysis largely aligned with the observed biodegradation pattern and showed that both core and transient microbiota members were able to detoxify NPA.

### NPA influences amino acid metabolism and transport in *Pantoea*

**Figure 2.**
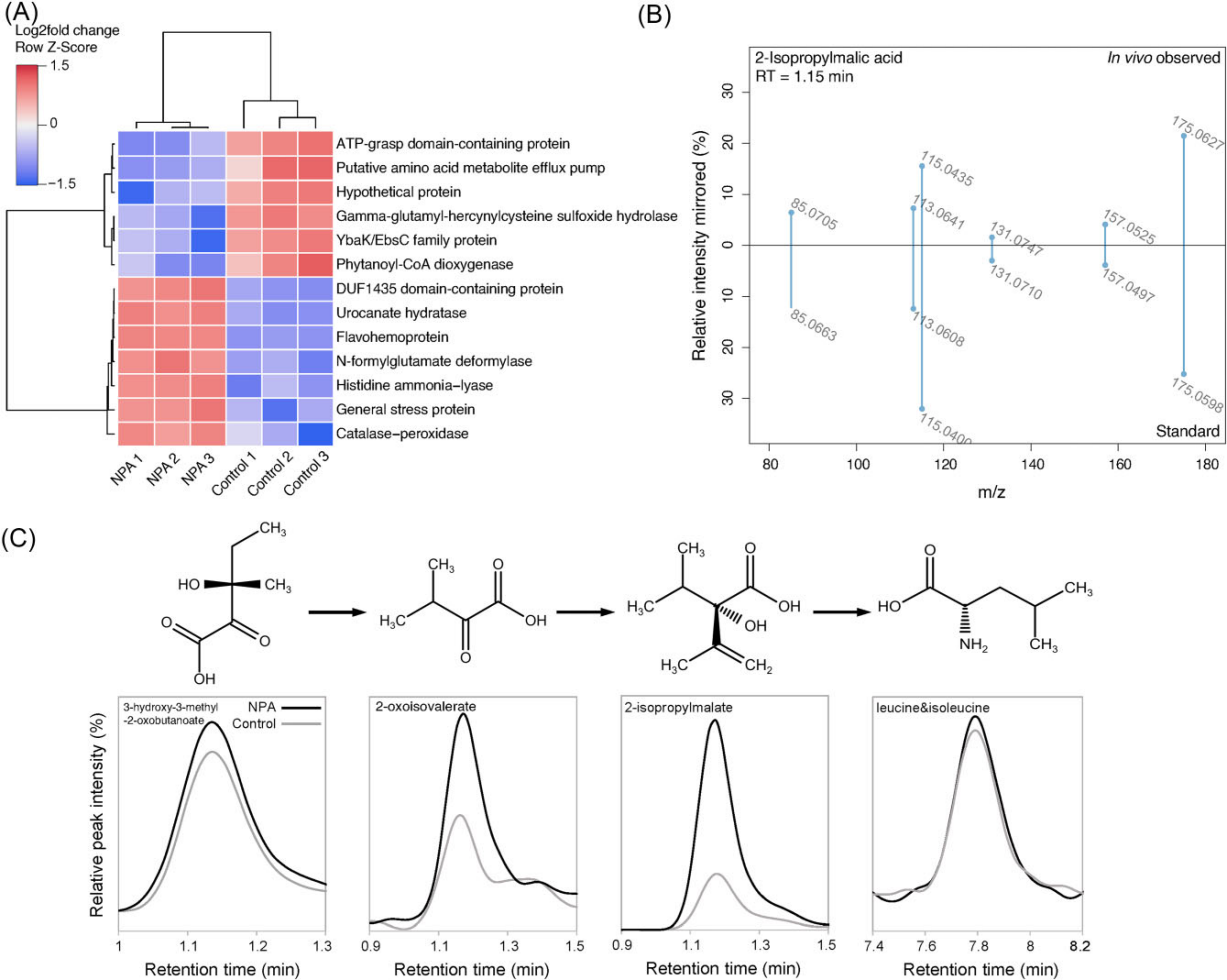
Transcriptome and metabolome of *Pantoea* sp. Nvir growing on M9 mineral medium with 100 µM NPA. (A) *Pantoea* sp. Nvir differential gene expression profile under growth with and without NPA. *Pantoea* was cultured in an M9 mineral salt medium supplemented with 100 µM NPA or an equimolar concentration of glycerol (control). Samples for RNA extraction (biological triplicates) were taken 2 h after NPA/glycerol supplementation. A heatmap shows differential gene expression (log_2_ fold change; cutoff = 2; *P* < .05) in *Pantoea* cultures. (B) Mirror plot of observed MS2 feature (top), and MS2 of a commercially purchased 2-isopropylmalic acid standard (bottom) analysed under the same experimental parameters with LC-qToF-MS. (C) Simplified l-leucine biosynthesis pathway with chemical structures and corresponding chromatograms at 6 h after NPA or glycerol (control) supplementation. 3-hydroxy-3-methyl-2-oxobutanoate and 2-oxoisovalerate were putatively identified based on m/z value (in italics), whereas 2-isopropylmalate and leucine/isoleucine were identified using chemical standards. Data are represented as the mean of biological quadruplicates.

To further investigate the influence of NPA on bacteria, we analysed the transcriptome of NPA-degrading *Pantoea* sp. Nvir, an obligate symbiont of *N. viridula* (Geerinck et al. 2022) (Fig. 2A). Our data demonstrated that under these stringent conditions, only 13 genes were differently expressed (Table S4). Two hours after the addition of NPA, when NPA was rapidly degraded, stress-response genes in *Pantoea* were differentially expressed, demonstrating its toxicity to the cell. Furthermore, NPA seemed to interfere with the oxidative stress response in *Pantoea*, since genes encoding gamma-glutamyl-hercynylcysteine sulfoxide hydrolase and phytanoyl-CoA dioxygenase were downregulated. In mycobacteria, the former is involved in the biosynthesis of the antioxidant metabolite ergothioneine and the latter is associated with a large diversity of oxidative reactions (Hausinger 2015). Interestingly, all downregulated genes (Fig. 2A) belong to a six-gene-long operon (genes 00010–00015). On the contrary, flavohemoprotein and catalase–peroxidase genes, encoding enzymes involved in NPA breakdown, were upregulated, supporting the observed NPA degradation *in vitro*. Surprisingly, no differential gene expression of the P3N monooxygenase gene was seen. The analysis showed *pno*A expression in both NPA and control samples (data not shown), suggesting that it might be constitutively expressed. Besides this, NPA influenced the expression of genes involved in amino acid metabolism and transport. Genes involved in the metabolism of histidine, encoding histidine ammonia-lyase and *N*-formylglutamate deformylase, were differentially expressed in cultures exposed to NPA. In contrast, the gene encoding YbaK/EbsC, a protein assumed to prevent translation by blocking the addition of an amino acid to a tRNA molecule, was downregulated in cultures exposed to NPA. The same was true for genes encoding a putative amino acid efflux pump, suggesting inhibition of amino acid biosynthesis and transport and thus possibly impaired supplementation of amino acids for the host.

In a proceeding experiment, we analysed the effect of NPA on the metabolome. Although NPA was rapidly degraded (Fig. 1B), no accumulation of succinate was observed in *Pantoea*, suggesting that NPA did not inhibit succinate dehydrogenase as suggested for eukaryotes (Bendiksen Skogvold et al. 2022). A similar observation was made in *Sodalis* cultures, which are not capable of NPA degradation (data not shown). Untargeted metabolomics did, however, reveal the accumulation of a metabolite with a m/z of 176.069 [M+H]^+^ in *Pantoea* cultures exposed to NPA. To identify this metabolite, we collected MS2 fragmentation spectra. When queried against metabolomics databases, the accumulating metabolite was putatively annotated as 2-isopropylmalate (2-IM). Subsequent comparison with a reference compound confirmed the identity of m/z 176.069 as 2-isopropylmalate (Fig. 2B). 2-IM is an intermediate in the biosynthesis pathway of l-leucine, in which it is synthesized from 2-oxoisovalerate by 2-isopropylmalate synthase and converted to 3-isopropylmalate by 3-isopropylmalate dehydratase (Strassman and Ceci 1963). Ultimately, l-leucine is formed through the activities of 3-isopropylmalate dehydrogenase and leucine transaminase. Figure 2(C) depicts a simplified l-leucine biosynthesis pathway in which we show that the concentration of intermediate metabolites, preceding the formation of l-leucine: 3-hydroxy-3-methyl-2-oxobutanoate (HMO), 2-oxoisovalerate (2-OV), and 2-IM increased in *Pantoea* cells during culturing with NPA (Hao et al. 2023). This results imply that NPA might have an inhibitory effect on 3-isopropylmalate dehydratase, dehydrogenase, or leucine transaminase by impairing the transformation of intermediate metabolites and l-leucine formation. Nevertheless, the l-leucine concentration in NPA and control samples were identical, with the constraint that leucine and isoleucine are isomers and were not chromatographically separated in our system. In conclusion, both the transcriptome and metabolome of *Pantoea* suggest that NPA might have an inhibitory effect on amino acid metabolism and transport, but further analysis of the effect of NPA on other bacteria is necessary to validate this hypothesis. Altogether, this shows that NPA, if not detoxified by other microbial community members, might impair amino acid supplementation by *Pantoea* to the host.

### Core microbiota inhibit the growth of transient bacteria

As a phloem feeder, *N. viridula*’s diet is nutritionally imbalanced. PS is rich in sucrose, the main sugar in PS (Amiard et al. 2004), and poor in amino acids and vitamins. Therefore, insects rely on their symbiotic microbial partners to supplement them with nutrients (Salem et al. 2014, Feng et al. 2019). Likewise, microbes that live in consortia to sustain growth, might depend on each other by biosynthesizing and exchanging certain metabolites (Zhang et al. 2018). Thus, we questioned whether any microbe–microbe interactions between members of the *N. viridula* gut community could lead to synergistic interactions. We determined that all strains favoured growth at 10% sucrose PS medium (Fig. S4). Depending on the plant species, the sucrose concentration varies considerably, however typically ranges from 10% to 30% (Broussard et al. 2023). Therefore, the artificially created PS medium reflected naturally present conditions and could be used to evaluate microbe–microbe interactions of gut bacteria *in vitro* and shed light on whether diet could shape gut microbial community.

**Figure 3.**
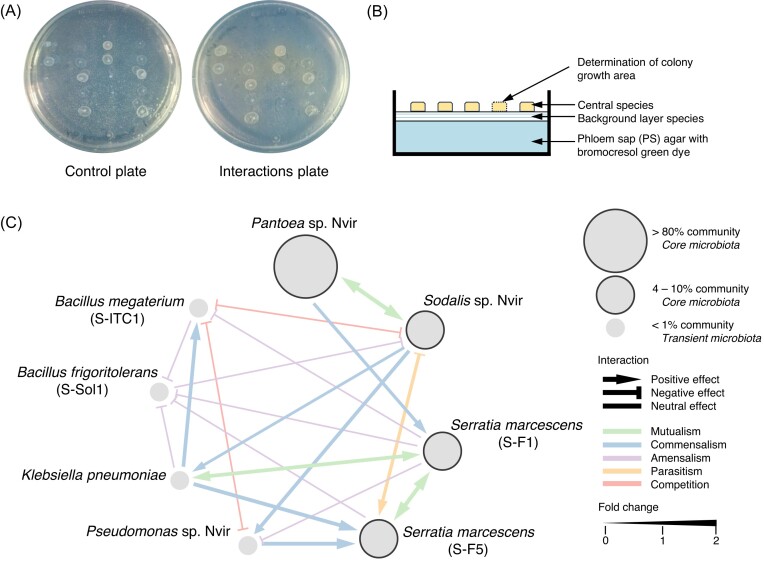
Interactions between *N. viridula* gut microbial community members on PS agar. (A) Monoculture control plate (no background layer species) and coculture binary interaction plate with background and central species. (B) Schematic representation of the method used to detect microbe–microbe interactions on PS medium. Each species was plated as a background layer on 10% PS medium containing bromocresol green dye and all central species were pinned on top. (C) Interaction network of core and transient gut microbiota of *N. viridula*. Node sizes represent the relative abundance of microbes in the gut calculated based on the metagenome data as previously described (Coolen et al. 2024); interactions are indicated with lines ended with arrows (positive effect), blunt arrows (negative effect), or lines (neutral effect); colour indicates the type of the relationship—mutualism, commensalism, amensalism, parasitism, and competition; line thickness reflects the strength of the interaction expressed as fold change calculated based on the difference of the growth area between interaction plate and control plate.

Next, we set out to identify pairwise interactions between gut species by comparing the performance of the cocultures against monocultures to identify interactions. Each strain (background layer species) was plated on a 10% sucrose PS agar as a lawn while all strains were pinned on top (central species). Changes in the colony size relative to the background layer were used to determine microbe–microbe interactions (Fig. 3A and B) (Blasche et al. 2021). The analysis revealed a dense interaction network between bacteria belonging to the core and transient microbiota (Fig. 3C), which included synergistic, negative, and neutral interactions. Remarkably, core microbiota had either mutualistic or commensal relationship, since *Pantoea* and *Sodalis* and both *Serratia* seemed to stimulate each other’s growth and *Pantoea* promoted the growth of *S. marcescens* (S-F1). The only negative interactions happened between *Sodalis* and *S. marcescens* (S-F5), where *Sodalis*’ growth was inhibited by *Serratia*. On the contrary, a substantial number of negative interactions were observed between the members of the transient and core microbiota. *Sodalis, S. marcescens* (S-F1), and *Pseudomonas* significantly inhibited the growth of *B. megaterium* while two *Serratia* strains, *Klebsiella* and *B. megaterium* negatively influenced *B. frigoritolerans. Klebsiella* is a transient bacterium whose growth was not negatively affected by either microbe but simultaneously contributed to promoting the growth of core and transient microbiota members. A total of five negative interactions were observed between core and transient bacteria, with four positive ones, out of which three were directed towards promoting the growth of *Serratia* strains. With this, we demonstrated that gut bacteria have complex relationships and interact with each other by possible exchange of metabolites, leading to the collaboration between core microbiota and competition between transient bacteria. Moreover, core microbiota suppressed transient microbes, suggesting that microbes contribute to shaping the insect’s gut microbial community, and thus explain the prevalence of certain microbes in the gut.

### Change of diet results in microbial community shift in the gut

**Figure 4.**
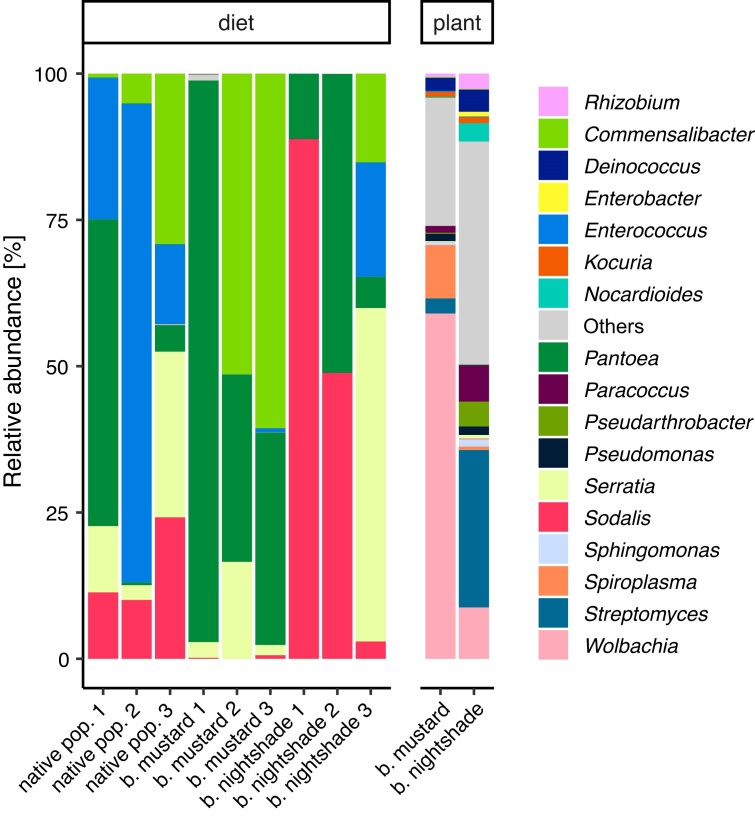
Influence of diet change on *N. viridula* gut microbial community composition. The samples were compared based on the 16S rRNA gene amplicon sequencing reads between adult *N. viridula* individuals (control; native population) feeding from black mustard, black nightshade, crown vetch, seeds of soybean, sunflower and black mustard, flat beans, and two populations which were fed for 6 months exclusively either on black mustard or black nightshade. Additionally, the plant microbiome of the plants was taken along as a control. The taxonomy is displayed at the genus level. Others represent the ASVs that average below 0.5% of all reads.

To ascertain whether diet influences the *N. viridula* gut microbial community composition, the microbiota of the native shield bug population was first determined by 16S rRNA gene profiling (Fig. 4). The results revealed the dominance of *Pantoea, Sodalis, Enterococcus, Serratia*, and *Commensalibacter* in the gut with the abundance of a particular genus differing between individual insects. Transfer of insects from the native population to single-plant diets was however only possible with black mustard and black nightshade. Insects did not establish a colony while feeding on crown vetch solely.

Switching to a single-plant diet resulted in a shift in the microbial community composition. In the *N. viridula* population reared on black mustard, the abundance of *Pantoea* and *Commensalibacter* in the gut microbial community increased in comparison to *N. viridula* grown with a multitude of plants. The *N. viridula* population feeding from black nightshade, in contrast, showed an increased relative abundance of *Sodalis*. Our genomic analysis indicated that *Sodalis* harboured genes encoding β-glucosidase and β-galactosidase and might therefore be capable of mediating the partial degradation of α-solanine (Hennessy et al. 2020). Interestingly, analysis of the plant microbiome indicated the presence of a highly abundant *Streptomyces* in black nightshade, known for its potent antibiotic production properties (Alam et al. 2022). Consequently, this presence could have led to the growth inhibition of various species within the *N. viridula* gut causing the shift of the gut microbial community. Moreover, the results demonstrated that the plant microbiome was different from the gut microbial community profile in any insect population, indicating that diet switch does not result in the acquisition of transient microbes, but rather in a shift in the abundance of already residing core symbionts.

Overall, our findings indicate that the gut microbial community of *N. viridula* is stable upon a change in dietary conditions which aligns with the earlier observations of the mutualistic interactions between core microbiota members and repression of transient bacteria. Taken that black mustard and black nightshade belong to distinct plant families and synthesize different secondary plant metabolites to deter insects (Fahey et al. 2001, Chowanski et al. 2016), *N. viridula* might favour symbionts through which detoxifying symbiosis could protect it against toxins or better fulfil nutritional requirements. In summary, regardless of the transition in diet, *N. viridula* maintains beneficial symbiotic microbes in the gut which likely fulfil detoxifying, digestive and nutritional functions.

## Discussion

In this study, we investigated the potential of the pest insect *N. viridula*’s core and transient microbiota to detoxify NPA using genomics, transcriptomics, metabolomics, and culturing approaches, and assessed how the host diet and microbes contribute to shaping insect gut microbial community. Endosymbiotic microorganisms associated with insects commonly contain a reduced genome when compared to their free-living counterparts (Hosokawa et al. 2006, Kikuchi et al. 2009, Manzano-Marin et al. 2015, Manzano-Marin and Latorre 2016). Interestingly, the genomes that were assessed here were obtained from symbionts that were capable of a free-living lifestyle and do not seem to have undergone a substantial genome reduction. Moreover, the genome presented here for the free-living *Pantoea* symbiont differs in size compared to the previously characterized *Pantoea* associated with *N. viridula* (Prado et al. 2009, Tada et al. 2011). Our previous study suggests that in fact several *Pantoea* symbionts in high abundance can be found in the *N. viridula* microbiota (Coolen et al. 2024) and further studies are required to disentangle their individual roles. The genome analysis of *N. viridula*-associated microbes revealed both host-specific adaptations, such as the ability to biosynthesize essential nutrients, as well as the adaptation to a free-living lifestyle by maintaining complete flagellar assembly (Yun et al. 2014). On the other hand, core and transient microbiota of *N. viridula* harboured large plasmids, which could allow bacteria and potentially their host to adapt to changing environmental conditions upon a diet switch to improve nutrient metabolism. Interestingly, as shown in another arthropod endosymbiont *Cardinium*, plasmids might also participate in the ongoing chromosomal genome reduction (Xiong et al. 2023), and thereby the presence of large plasmids might reflect the beginning of an ongoing genome reduction among *N. viridula* microbial community members (Campbell et al. 2015, 2017, Van Leuven et al. 2014).

The presence of monooxygenases in bacterial genomes would also suggest that the *N. viridula* gut is oxic. Our previous results yielded a comparable number of aerobic as anaerobing microorganisms during cultivation indicating that the microbiome is not composed of strict but rather facultative aerobes (Coolen et al. 2024). Moreover, the short food retention time of piercing and sucking insects further supports the presumption of an oxic environment in the gut, as rapid transit times reduce the development of anaerobic conditions (Al-Snafi 2016, Coolen et al. 2022). Considering detoxification capabilities the oxic NPA degradation likely takes place in *N. viridula*’s gut. What is more, the genes encoding for enzymes involved in the degradation of NPA were widespread among core and transient microbial community members. This was in accordance with the observed biodegradation capacity of six out of eight *N. viridula* microbial community members, which could benefit the host and nondetoxifying microbial community members with protection against this toxic secondary plant metabolite. Transient microbes, however, grew poorly in cocultures, and therefore it is unlikely that they benefit *N. viridula* with their NPA degradation capacity. In light of the development of novel pest management strategies, this knowledge could be used to target toxin-degrading microbes via the elimination of the detoxifying genes with the CRISPR-Cas9 mechanism, which would decrease the spread of toxin resistance phenotype among insects (Sander and Joung 2014, Selle and Barrangou 2015, Zhao et al. 2020, Rogowska-van der Molen et al. 2023). Furthermore, NPA was previously shown to cause irreversible inhibition of the succinate dehydrogenase enzyme in the TCA cycle in eukaryotes and thus accumulation of succinate (Bendiksen Nishino et al. 2010, Rogowska-van der Molen et al. 2022, Skogvold et al. 2022), but a similar observation was not made in the prokaryotes *Pantoea* or *Sodalis*, demonstrating that NPA does not impair the functioning of the TCA cycle in bacteria but rather affects amino acids metabolism and transport. Particularly l-leucine biosynthesis seemed to be inhibited by NPA since it caused intracellular accumulation of l-leucine biosynthesis intermediates. Whether the accumulation of intermediate metabolites caused toxicity to the cells requires further validation.

Members of the microbial community found in *N. viridula* were also shown to interact with one another, shaping the gut microbiota. We unravelled microbial interactions between *N. viridula* gut microbiota members and demonstrated that in an artificial PS medium bacteria formed a complex community network. A clear relationship was observed between two major *N. viridula* symbionts, *Sodalis* and *Pantoea*, which had a mutualistic relationship and promoted each other’s growth in the sugar-rich and nutrient-deficient PS medium. A similar tendency was shown in facultative *Serratia* symbionts. These findings suggest a metabolite exchange via cross-feeding between bacteria and indicate the adaptations of core microbes to living in proximity (Blasche et al. 2017, 2021, Goldford et al. 2018). While core microbiota thrived in the PS medium, the growth of bacteria associated with the transient microbiota was largely inhibited by either transient or core microbiota implying a lack of adaptive ability to sustain growth under nutrient-deficient conditions (Yun et al. 2014). Altogether, our results indicate that both diet and microbial interactions contribute to shaping the host-associated microbial community and with that also its detoxifying symbiosis potential. This dynamic between *N. viridula* microbiota members demonstrated the colonization resistance of core microbes against nonsymbiotic bacteria, preventing the disturbance of the gut community.

Diet is another important factor shaping and influencing an insect’s phenotype and gut bacterial community (Colman et al. 2012, Amato et al. 2019, Luo et al. 2021). Our results demonstrate that switching from a polyphagous to a single-plant diet caused a shift in core microbial community composition and underscored the dissimilarity to the plant microbiota. These findings imply an overall stability of the core gut microbiome in response to dietary shifts. Likewise, similar patterns were reported by Medina et al. (2018) who observed a shift in the relative abundance of *Pantoea, Yokenella*, and *Enterococcus*, belonging to the core microbiota, in *N. viridula* during the transition between host plants. Tinker and Ottesen (2016) investigating the gut microbiota of the American cockroach *Periplaneta americana* also found a stable core microbial community in response to diet change. The presence of a consistent core microbiota in *N. viridula* suggests that the symbionts are vital to the hosts’ fitness (Sudakaran et al. 2015). Analysis of the metabolic potential of *N. viridula*-associated microbiota confirmed the ability to biosynthesize essential amino acids and B vitamins fulfilling hosts’ nutritional requirements. Concordantly, several studies reported that the experimental removal of microbiota by egg-surface sterilization negatively affected *N. viridula* fitness and survival (Prado et al. 2009, Tada et al. 2011). On the other hand, we observed that the plant microbiome differed substantially from the gut microbial community implying the selective passing of microbes and suggesting that the selection mechanism could discriminate the core symbionts from transient, nonsymbiotic bacteria (Ohbayashi et al. 2015). One such mechanism could be microbe–microbe interactions as seen in the pairwise interactions experiment, which demonstrated the mutualistic interactions between core microbiota members and repression of transient bacteria. On the other hand, as described by Kim et al. (2013) insect midgut epithelia could produce antimicrobial substances and in that way control the selective infection of symbionts to the M4 midgut crypts. Taken together, these findings highlight the complex dynamics behind the microbial colonization of the insect gut tract.

While *N. viridula* feeds on a polyphagous diet, transitioning to a single-plant source could pose challenges for polyphagous insects that lack adaptation for a single-plant species diet (Bernays and Chapman 2007). We observed that *N. viridula* insects established colonies on black mustard and black nightshade but were not capable of feeding solely with crown vetch. A recent study indicated that feeding on various plants provides insects with a nutritional balance and reduces the intake of toxic compounds from individual plant species (Friedrichs et al. 2022), thus feeding on crown vetch solely might have caused limited nutrient availability as compared to black mustard and black nightshade. While our data demonstrated the ability of *N. viridula* microbiota to degrade NPA, the short food retention time and high concentration of NPA in crown vetch (Al-Snafi 2016, Coolen et al. 2022) suggest that the microbial population might not have sufficient time to degrade the toxin and protect the host or nondetoxifying symbionts. Nevertheless, when comparing the gut microbial composition of black mustard and black nightshade populations a shift in gut microbial relative abundance was noted. As previously shown in camellia weevils, this variance could potentially result from the production of distinct toxins by the plants to deter insects (Zhang et al. 2020). Black nightshade is known for the production of solanaceous toxins α-solanine and α-chaconine and the observed increase in *Sodalis* abundance could be linked with its capability to degrade toxins (Mohy-ud-Din et al. 2010). The genomic analysis revealed that *Sodalis* encoded β-glucosidase and β-galactosidase, which could partially degrade α-solanine. Although *Sodalis* lacked α-rhamnosidase, a stepwise removal of glucose and galactose from α-solanine’s sidechains was shown to substantially decrease its toxicity (Jensen et al. 2009a,b). On the contrary, black mustard produces ITC to deter insects that are liberated by the glucosinolate–myrosinase defence system (Winde and Wittstock 2011). Even though ITCs are well-studied, the microbial detoxification pathway of most ITCs remains unclear, making it challenging to deduct from our genomic data whether *N. viridula* microbiota has the metabolic potential to facilitate ITC degradation. In summary, the results suggested that regardless of dietary shifts, *N. viridula* can maintain core microbes, which in return could mediate the detoxification of plant toxins and participate in digestion and nutrient supplementation.

In conclusion, our study revealed that the core microbiota of *N. viridula* exhibits signs of ongoing genome reduction by harbouring large plasmids and host-specific adaptation to symbiotic lifestyles. Gut-associated bacteria had varying resistance to NPA and the obligate *Pantoea* symbiont, and facultative *Serratia* strains and other transient bacteria, could rapidly degrade NPA, possibly providing *N. viridula* with resistance to plant defences. We also showed a possible new mechanism of action of NPA in bacteria by demonstrating that cultivating *Pantoea* with NPA blocks the biosynthesis pathway of l-leucine and causes the accumulation of 2-isopropylmalate. Further, our findings revealed that the *N. viridula* microbiota is stable in response to diet change and that microbe–microbe interactions participate in shaping gut bacterial community composition. Taken together, this research highlights the importance of studying insect–plant–microbe interactions to obtain fundamental knowledge on a tritrophic level, which could lead to the elucidation of sustainable pest management strategies in the future.

## Supplementary Material

fiae150_Supplemental_Files

## Data Availability

The transcriptome, genomes, and plasmids sequencing data are deposited in the European Nucleotide Archive under Project Number PRJEB70466 and submission ERA27452063.
