# Supplementary material for: Unveiling detoxifying symbiosis and dietary influence on the Southern green shield bug microbiota
Source: FEMS Microbiol Ecol. 2024 Nov 7;100(12):fiae150. doi: 10.1093/femsec/fiae150 (PMC11585277; doi:10.1093/femsec/fiae150)
Supplement: fiae150_Supplemental_Files [file fiae150_supplemental_files.zip › Supplementary_material_Microbial-dynamics.pdf]

Supplementary Material

The supplementary material includes Table S1-S3 and Figures S1-S6

Supplementary Table S4 is supplied individually as excel file.

Table S1. Metabolic potential to biosynthesize essential amino acids and B vitamins. "+" indicates the ability to produce and "-" the lack of the ability to produce a metabolite, based on genome analysis and presence/absence of respective genes encoding for biosynthetic proteins. Metabolic pathway analysis was conducted in KEGG.

**Essential amino acids**	***Pantoea* sp. Nvir**	***Sodalis* sp. Nvir**	***Serratia marcescens* (S-F1)**	***Serratia marcescens* (S-F5)**	***Bacillus frigoritolerans* (S-Sol)**	***Pseudomonas* sp. Nvir**	***Klebsiella pneumoniae***	***Bacillus megaterium* (S-ITC1)**
**valine**	+	+	+	+	+	+	+	+
**leucine**	+	+	+	+	+	+	+	+
**isoleucine**	+	+	+	+	+	+	+	+
**threonine**	+	+	+	+	+	+	+	+
**methionine**	+	+	+	+	+	-	+	+
**arginine**	+	+	+	+	+	+	+	+
**lysine**	+	+	+	+	+	+	+	+
**histidine**	+	+	+	+	+	-	+	+
**phenylalanine**	+	+	+	+	-	+	+	-
**tryptophan**	+	+	+	+	+	+	+	+
**Vitamins**								
**vitamin B1 (thiamine)**	+	+	+	+	+	+	+	-
**vitamin B2 (riboflavin)**	+	+	+	+	+	+	+	+
**vitamin B5 (pantothenic acid)**	+	+	+	+	+	+	+	+
**vitamin B6 (pyridoxine)**	+	-	+	+	+	+	+	+
**vitamin B7 (biotin)**	-	-	-	-	-	-	-	-
**vitamin B9 (folate)**	+	+	+	+	-	-	+	+

Table S2. Microbial metabolic potential to degrade toxic secondary plant metabolites.

Metabolite	Enzyme	***Pantoea* sp. Nvir**	***Sodalis* sp. Nvir**	***Serratia marcescens* (S-F1)**	***Serratia marcescens* (S-F5)**	***Bacillus frigoritolerans* (S-Sol)**	***Pseudomonas* sp. Nvir**	***Klebsiella pneumoniae***	***Bacillus megaterium* (S-ITC1)**
**NPA**	Nitronate monooxygenase	*pno*A	-	*nmo*A	*nmo*A	*pno*A	*pno*A *pnm*R	*pno*A	*nmo*A
**α-solanine α-chaconine**	α-rhamnosidase	-	-	-	-	-	-	-	-
	β-glucosidase	-	*bgl*B	-	-	-	-	-	-
	β-galactosidase	*lac*Z	*lac*Z	*ebg*A	*ebg*A *lac*Z	-	-	*lac*Z	*bga*A *cbg*A
**ITC**	Isothiocyanate hydrolase	-	-	-	-	-	-	-	-

Table S3. Characterization of bacteria isolated from the *N. viridula* microbiome to resist 3-nitropropionic acid – raw data. Bacterial gut isolates were tested towards their resistance to NPA in disc diffusion assays with increasing NPA concentration (0 – 1000 mM) applied to the paper discs (6 mm Ø). The diameter of inhibition halo is expressed in mm per replicate. Paper discs have a 6 mm diameter, therefore area of inhibition cannot be below 6 mm.

Pantoea* sp. *Nvir				
	Inhibition zone (mm)			
NPA Concentration (mM)	Replicate 1	Replicate 2	Average	St. dev.
0	6	6	6	0.000
93.75	6	6	6	0.000
187.5	7	8	7.5	0.707
375	7	8	7.5	0.707
750	9	8	8.5	0.707
1000	10	9	9.5	0.707
Sodalis* sp. *Nvir				
	Inhibition zone (mm)			
NPA Concentration (mM)	Replicate 1	Replicate 2	Average	St. dev.
0	6	6	6	0.000
93.75	6	6	6	0.000
187.5	9	11	10	1.414
375	16	14	15	1.414
750	20	18	19	1.414
1000	27	26	26.5	0.707
***Serratia marcescens* (S-F1)**				
	Inhibition zone (mm)			
NPA Concentration (mM)	Replicate 1	Replicate 2	Average	St. dev.
0	6	6	6	0.000
93.75	6	6	6	0.000
187.5	6	6	6	0.000
375	8	7	7.5	0.707
750	7	7	7	0.000
1000	8	7	7.5	0.707
***Bacillus frigoritolerans* (S-Sol1)**				
	Inhibition zone (mm)			
NPA Concentration (mM)	Replicate 1	Replicate 2	Average	St. dev.
0	6	6	6	0.000
93.75	6	7	6.5	0.707
187.5	7	7	7	0.000
375	9	9	9	0.000
750	10	11	10.5	0.707
1000	12	12	12	0.000
Klebsiella pneumoniae				
	Inhibition zone (mm)			

NPA Concentration (mM)	Replicate 1	Replicate 2	Average	St. dev.
0	6	6	6	0.000
94	6	6	6	0.000
188	6	6	6	0.000
375	6.5	6	6.25	0.354
750	8	7	7.5	0.707
1000	8.5	7	7.75	1.061

***Pseudomonas* sp. Nvir**

	Inhibition zone (mm)			
NPA Concentration (mM)	Replicate 1	Replicate 2	Average	St. dev.
0	6	6	6	0.000
94	6.5	6.5	6.5	0.000
188	6.5	6.5	6.5	0.000
375	7	7	7	0.000
750	9	9	9	0.000
1000	10	11	10.5	0.707

***Serratia marcescens* (S-F5)**

	Inhibition zone (mm)			
NPA Concentration (mM)	Replicate 1	Replicate 2	Average	St. dev.
0	6	6	6	0.000
94	7	6	6.5	0.707
188	7	7	7	0.000
375	7	6	6.5	0.707
750	7	6	6.5	0.707
1000	7.5	8	7.75	0.354

***Bacillus megaterium* (S-ITC1)**

	Inhibition zone (mm)			
NPA Concentration (mM)	Replicate 1	Replicate 2	Average	St. dev.
0	6	6	6	0.000
94	6	6	6	0.000
188	6	6	6	0.000
375	6	6	6	0.000
750	11	12	11.5	0.707
1000	12	12	12	0.000

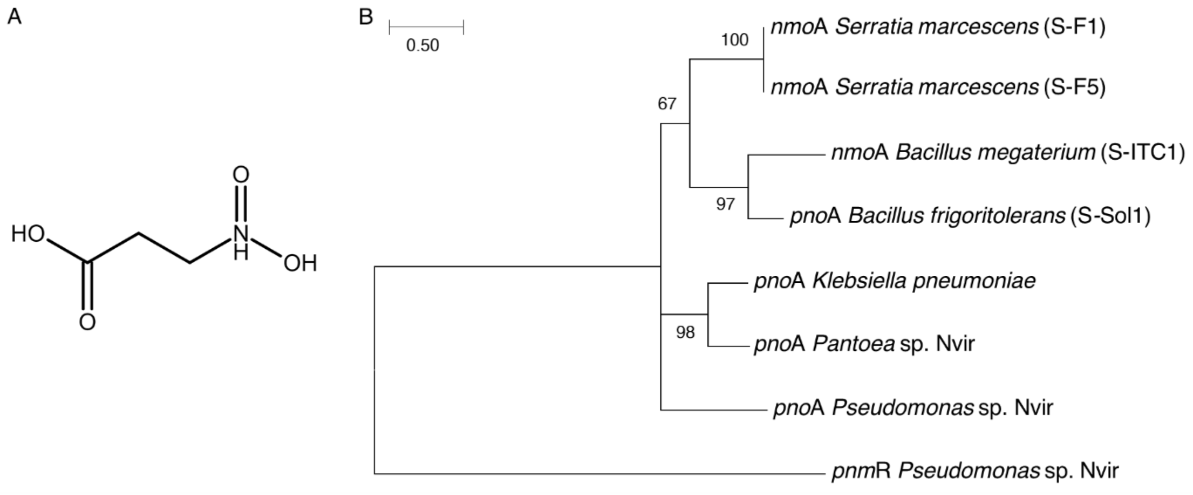

Figure S1. Evolutionary assessment of genes encoding NPA-degrading enzymes found in genomes of *N. viridula* microbiota. A) Chemical structure of 3-nitropropionic acid. **B)** Neighbour-joining tree of *pno*A, *nmo*A and *pnm*R NPA-degrading genes found in *N. viridula* microbiota. Amino acid sequences were aligned with MUSCLE (Edgar, 2004), and a phylogenetic tree was calculated with MEGA7 (Kumar et al., 2016) using 500 bootstraps. Branches' values are the percentages of the replicate tree in which the associated genes clustered together in the bootstrap test (≥50). Branches are drawn to scale.

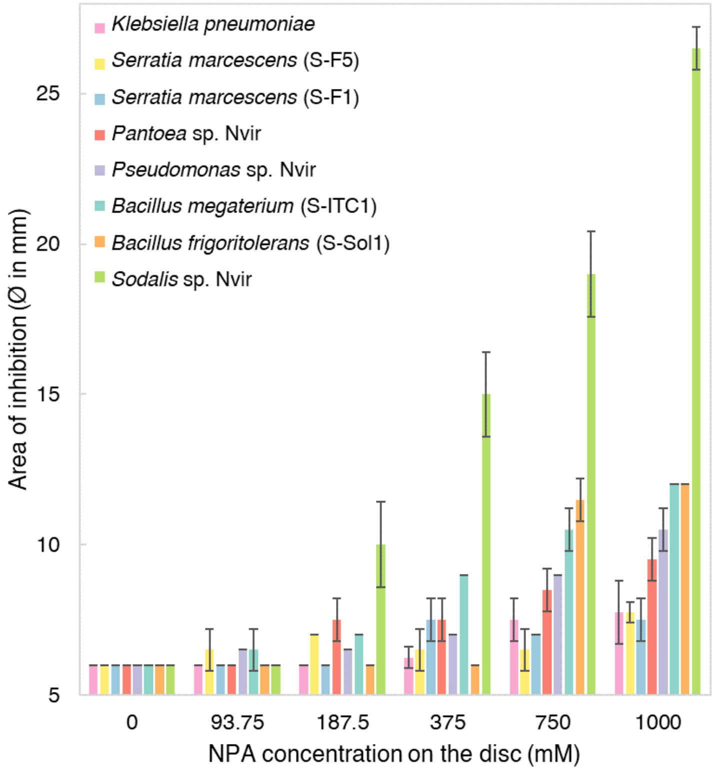

Figure S2. Characterization of bacteria isolated from the *N. viridula* microbiome to resist 3-nitropropionic acid. Bacterial gut isolates were tested towards their resistance to NPA in disc diffusion assays with increasing NPA concentration (0 – 1000 mM) applied to the paper discs (6 mm Ø). The diameter of inhibition halo is expressed in mm ± standard deviation (n = 2). Paper discs have a 6 mm diameter, therefore area of inhibition cannot be below 6 mm.

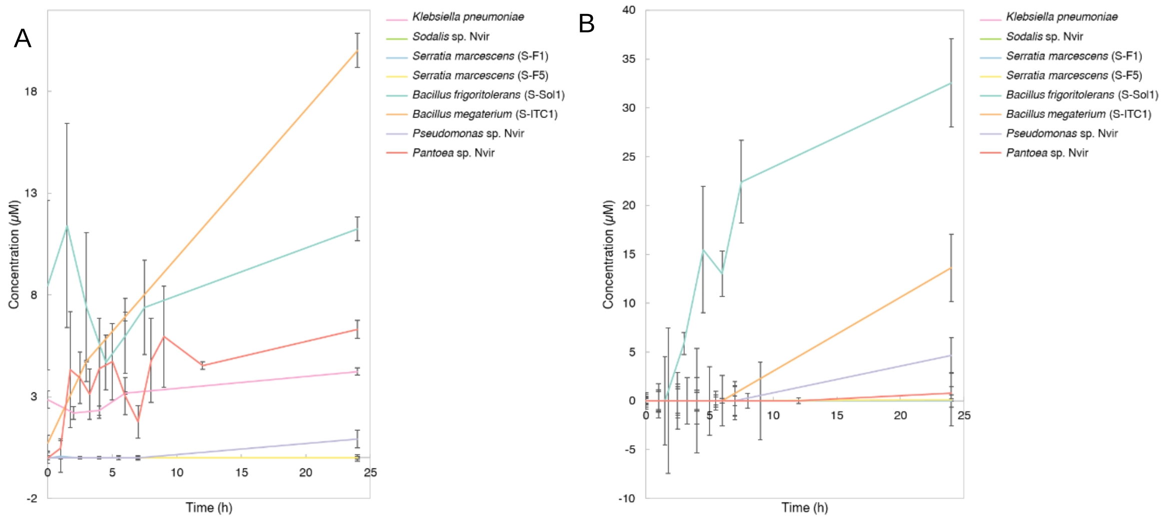

Figure S3. Nitrite and nitrate profiles in bacterial cultures isolated from *N. viridula*. A) Profile of nitrite during 24 h incubation in M9 mineral salt medium with 100 µM NPA. B) Profile of nitrate during 24 h incubation in M9 mineral salt medium with 100 µM NPA. Data are represented as mean ± standard error (n = 3).

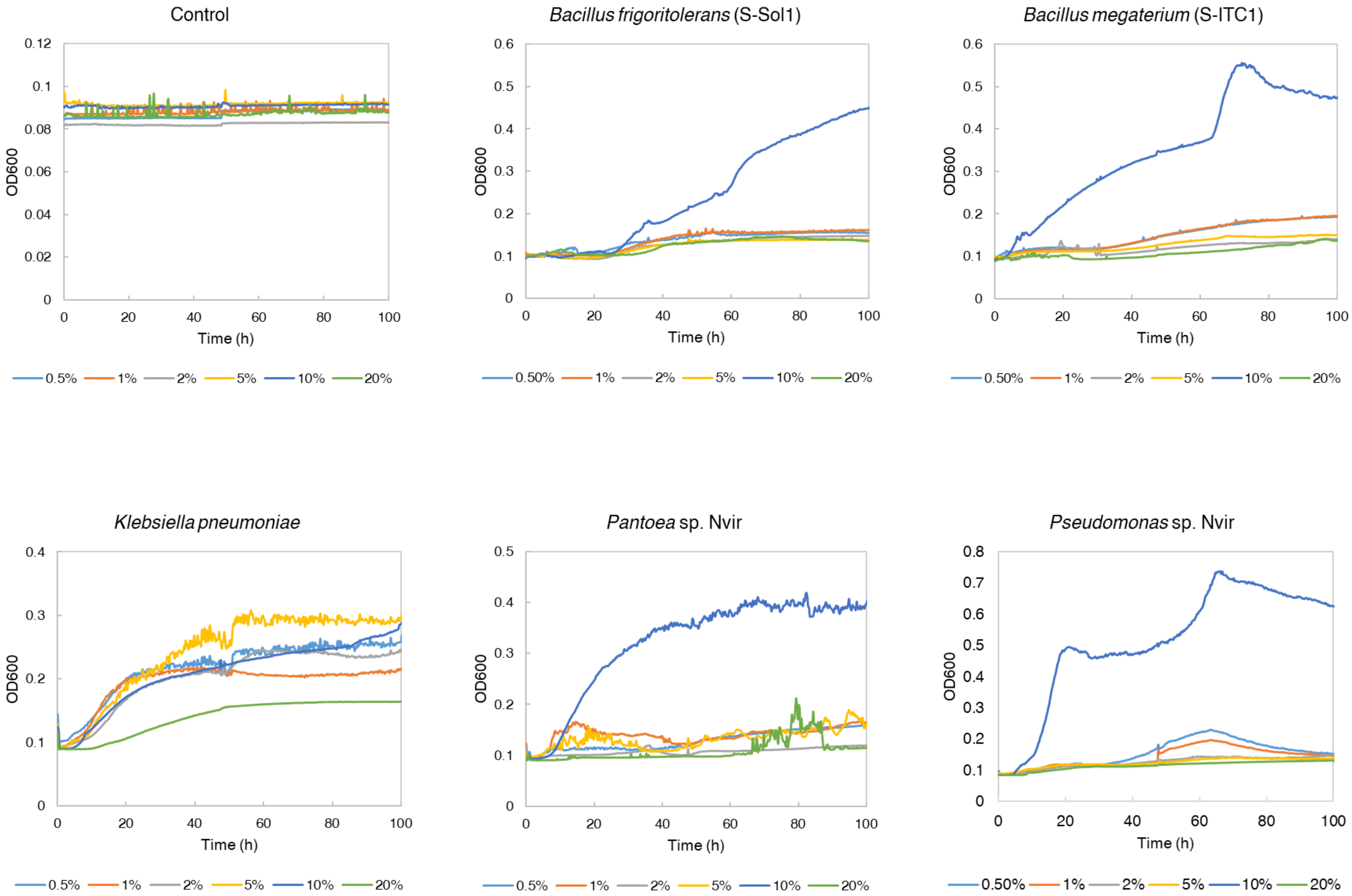

Control
Bacillus frigoritolerans (S-Sol1)
Bacillus megaterium (S-ITC1)
Klebsiella pneumoniae
Pantoea sp. Nvir
Pseudomonas sp. Nvir
OD600
Time (h)
0.5% 1% 2% 5% 10% 20%
0.50% 1% 2% 5% 10% 20%

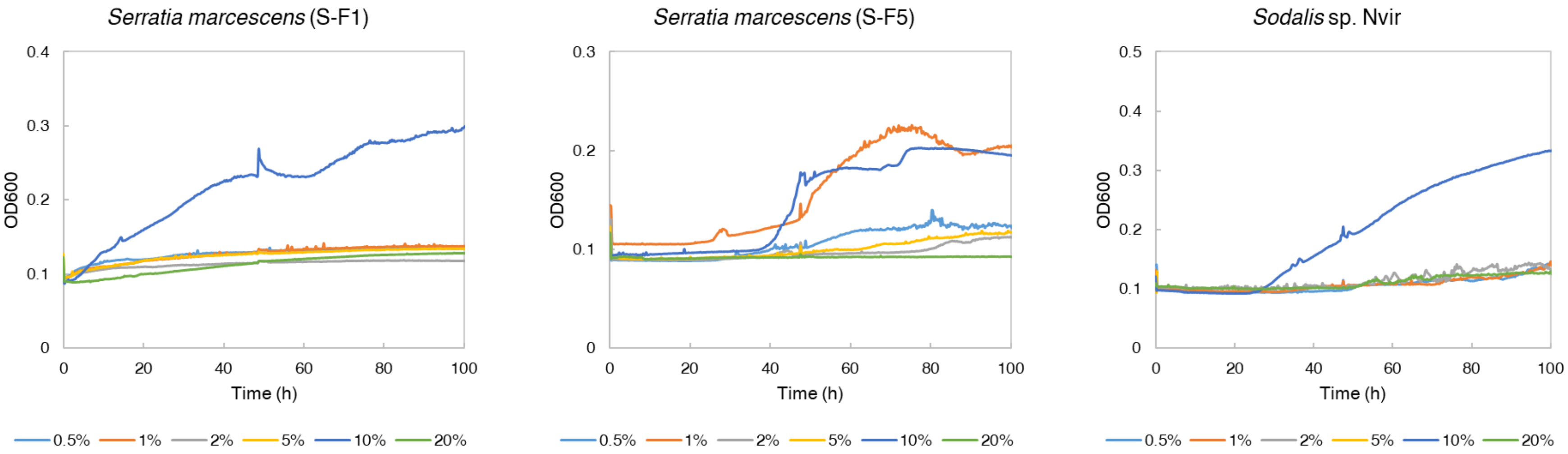

Figure S4. Growth curves of eight bacterial strains isolated from *N. viridula*. Growth was compared in phloem-sap (PS) medium with different sucrose concentrations – 0, 0.5%, 1%, 2%, 5%, 10% and 20% and monitored by measuring OD_{600} every 15 min for 100h. Bacterial cultures were inoculated at the initial OD_{600} of 0.05 (n = 3; technical replicates). Control is an uninoculated PS medium.

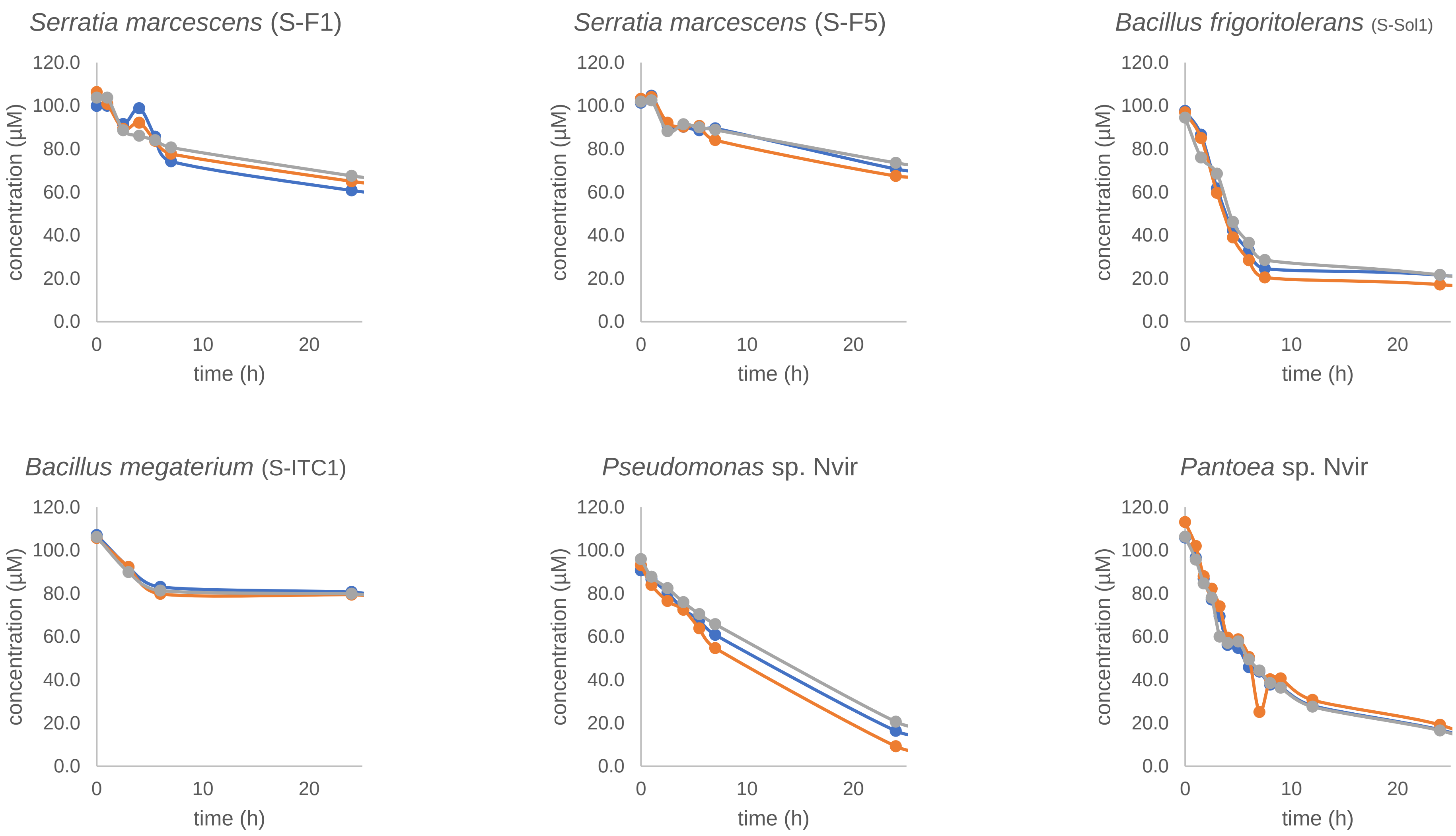

Serratia marcescens (S-F1)
Serratia marcescens (S-F5)
Bacillus frigoritolerans (S-Sol1)
Bacillus megaterium (S-ITC1)
Pseudomonas sp. Nvir
Pantoea sp. Nvir
concentration (µM)
time (h)
120.0
100.0
80.0
60.0
40.0
20.0
0.0
0
10
20

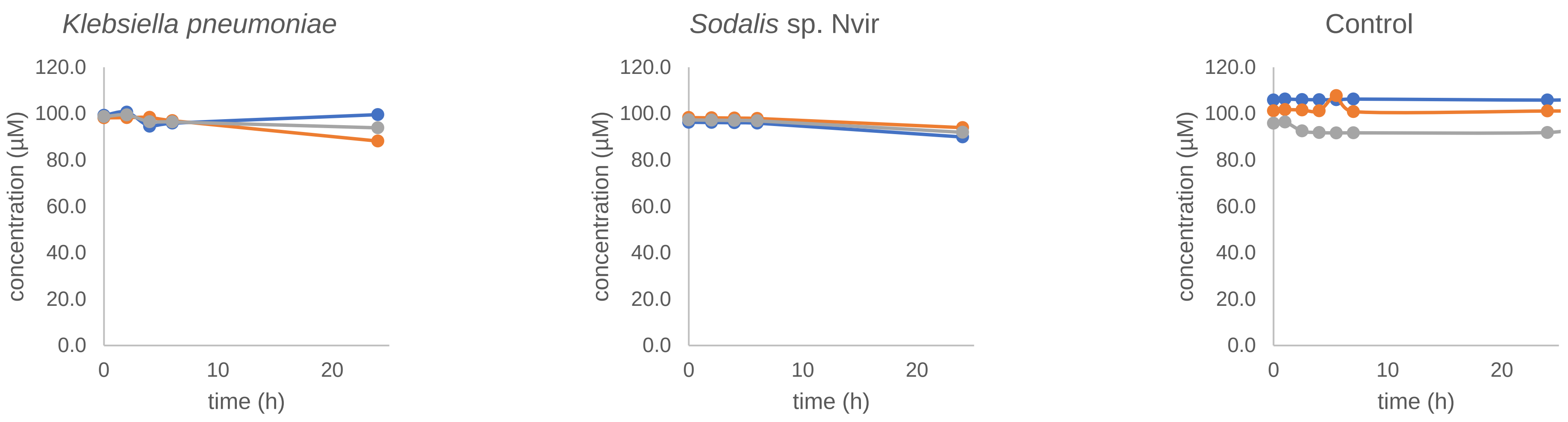

Figure S5. Characterization of bacteria isolated from the *N. viridula* microbiome to resist and detoxify 3-nitropropionic acid. Bacterial gut isolates were incubated in 50 mL M9 mineral salt medium supplemented with 100 µM NPA and detoxification was monitored for 24 h. Control represents uninoculated M9 medium containing NPA. NPA concentrations were measured by HPLC. Data are represented as individual biological replicates.

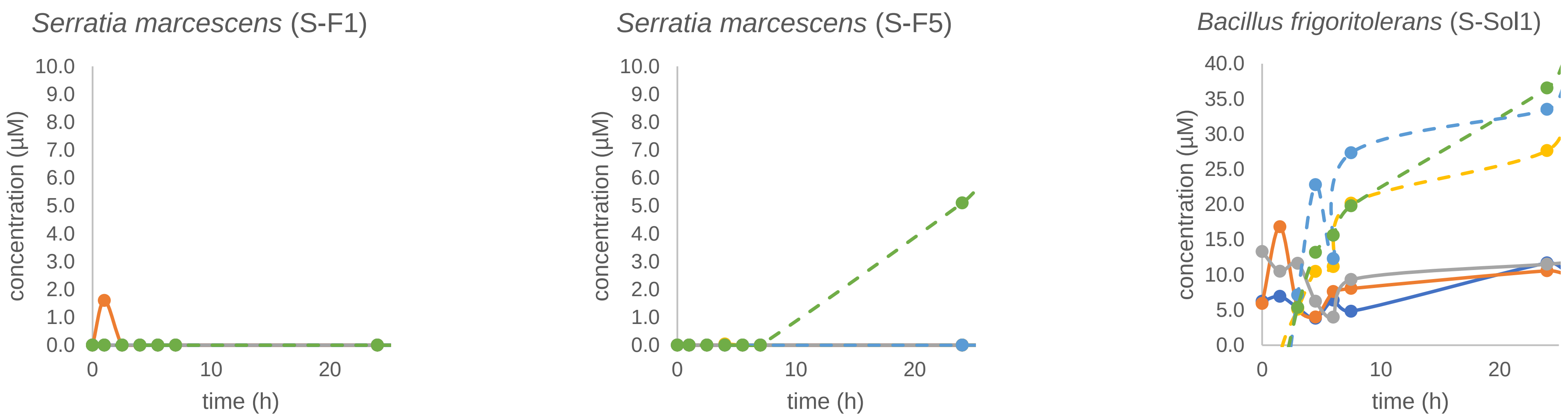

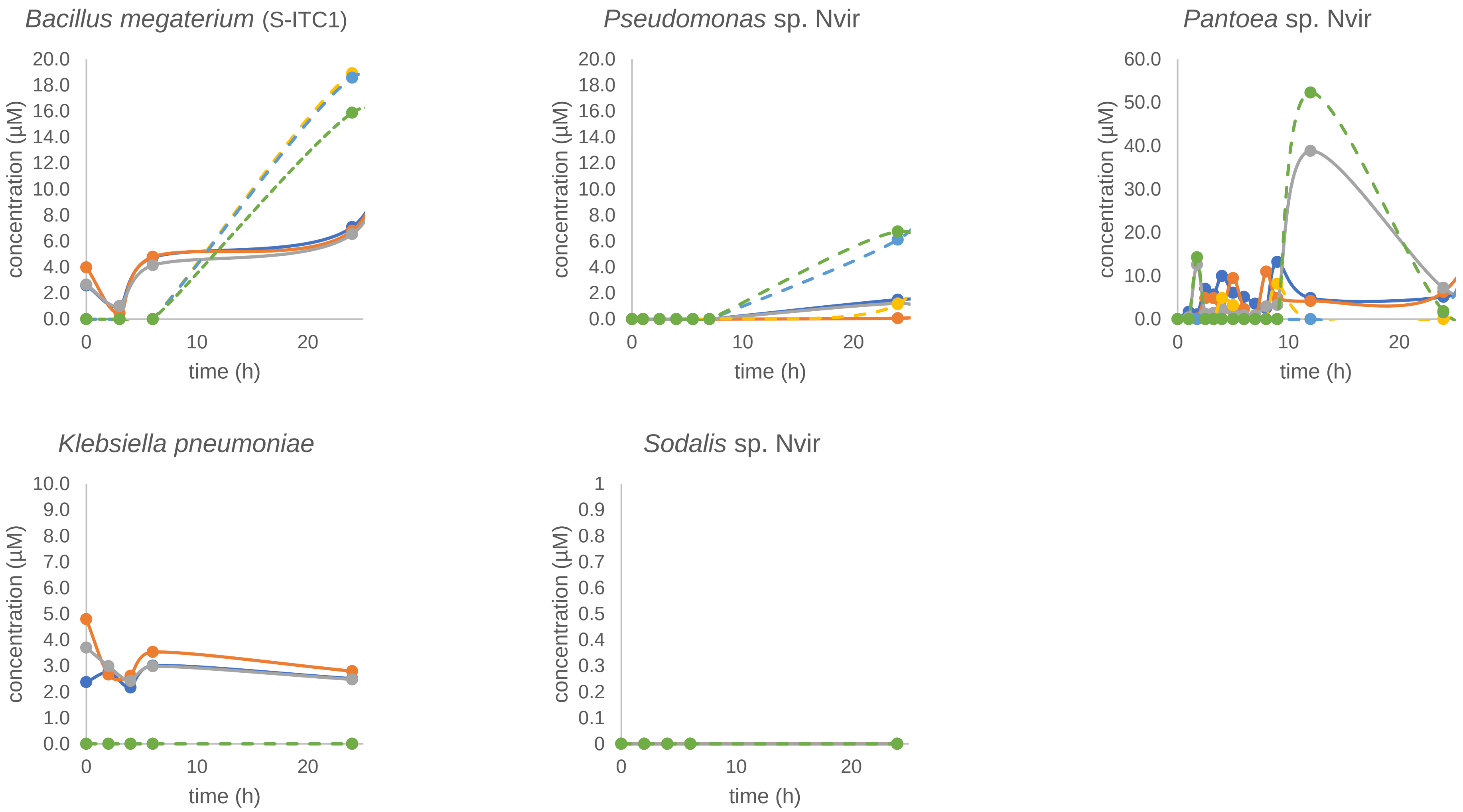

Figure S6. Nitrite and nitrate profiles in bacterial cultures isolated from *N. viridula*. Profiles of nitrite (solid lines; grey, dark blue, orange) and nitrate (dashed lines; green, light blue, yellow) during 24 h incubation in M9 mineral salt medium with 100 µM NPA. Data are represented as individual biological replicates.
